# A Rare Case of Hemoperitoneum Diagnosed with Point of Care Ultrasound (POCUS) 

**DOI:** 10.24908/pocus.v9i2.17611

**Published:** 2024-11-15

**Authors:** Jina Bai, Todd Cutler

**Affiliations:** 1 Department of General Internal Medicine, Section of Hospital Medicine, Weill Cornell Medical Center NYC, NY USA

**Keywords:** POCUS, Paracentesis, Abdominal Point-of-Care Ultrasound Case presentation, Hemoperitoneum

## Abstract

A 68-year-old man presented with one week of vague abdominal symptoms and was found to have new ascites and pulmonary embolism for which a heparin drip was initiated. We report a case diagnosing hemoperitoneum with point of care ultrasound (POCUS). Identifying hemoperitoneum can be challenging, but POCUS can be a useful tool for its diagnosis. There is limited literature on the sonographic characteristics of hemoperitoneum. Echogenicity of fluid is not a reliable distinguisher between exudative and transudative effusions. The diagnosis of hemoperitoneum in this case was suggested by the progression of POCUS findings over time rather than sonographic characteristics by themselves.

## Case presentation

A 68-year-old man with a history of Roux-en-Y bariatric surgery presented to the hospital with one week of nausea, vomiting, diarrhea, and abdominal pain. On examination, the patient was in sinus tachycardia at 122 beats per minute. His laboratory work showed leukocytosis, a hemoglobin of 12.5 g/dL, and a lipase of 857 U/L. Computed tomography (CT) of the abdomen and pelvis showed gastric bypass anatomy and new small to moderate ascites, as well as segmental and subsegmental thrombi at the bases of both lungs. He was started on a heparin drip for treatment of acute pulmonary embolism. The patient was admitted for the management of acute pulmonary embolism, presumed pancreatitis given abdominal pain and elevated lipase, and ascites of unknown etiology.

A POCUS examination of the abdomen was performed (Video S1), with the probe positioned coronally in the right midaxillary line at the level of umbilicus, which showed simple appearing ascites with some floating particles. Heparin drip was held for an hour in anticipation of paracentesis. After an hour, POCUS was performed again immediately prior to paracentesis in the same probe position as previously scanned (Video S2), which demonstrated a new partially solid, hyperechoic and heterogeneous collagenous material adjacent to the liver. A diagnostic paracentesis confirmed the presence of blood with an ascitic fluid hematocrit of 11 g/dL. 

A CT angiogram revealed hyperattenuating material near the gallbladder fossa, consistent with hemoperitoneum although no active bleeding was identified. His hemoglobin continued to decline (Table 1) and the patient received transfusions. An inferior vena cava (IVC) filter was placed and, following a period of monitoring, therapeutic anticoagulation was cautiously resumed and the patient was discharged home. The patient was subsequently readmitted with hematochezia but esophagogastroduodenoscopy (EGD), colonoscopy, and capsule endoscopy failed to identify a source of bleeding. After two weeks, during which the patient received nine units of packed red blood cells, an exploratory laparoscopy revealed hemorrhagic ascites and a posterior perforation in the bypassed duodenum with surrounding inflammatory changes abutting the gallbladder, liver, and the hepatic flexure of the colon. This perforation was likely the cause of the initial abdominal pain on admission and the subsequent source of bleeding.

## Discussion

Hemoperitoneum is an accumulation of blood in the peritoneal cavity which can be caused by various factors, including complications from anticoagulation. It is difficult to diagnose hemoperitoneum with POCUS alone 1. There are no sonographic findings that can definitively identify active or prior bleeding within effusions or ascites. Plankton sign and hematocrit sign are sometimes cited to suggest bleeding 2, 3. The plankton sign refers to hyperechoic swirling debris within a fluid space and has been described in infectious and malignant effusions as well as in acute bleeding 2, 3, 4. However, even transudative effusions may appear to have swirling echogenicity 2, 4, 5, 6. In a study by Chen et al., only 45% of transudative pleural effusions had an anechoic sonographic appearance, while the remaining 55% had a complex non-septated sonographic appearance 6. It is likely that effusions contain components such as cells, proteins, and lipids, which can be seen as echogenic hyperechoic particles. The hematocrit sign refers to the development of a gravitationally dependent fluid-debris level within an effusion 4; however, as in our patient, it is not always present and therefore has unreliable sensitivity for acute bleeding 7, 8. Other sonographic features such as heterogeneous echogenicity, fibrin strands, and septations can be seen in effusions due to infection, malignancy, and bleeding.

**Table 1:** Initial hemoglobin trend

Time Frame	Hemoglobin (g/dL)
At admission	12.5
HD 1 AM after initiation of heparin	11.7
HD 1 PM at the time of paracentesis	10.0
HD 2	8.2 *Transfused
HD 3	7.5

HD, Hospital day.

The most researched area of hemoperitoneum is in trauma. The focused assessment with sonography in trauma (FAST) exam is extensively described in the literature for detecting blood in the abdomen following blunt trauma 7, 8. There is less literature on how active bleeding appears and changes over-time within pre-existing fluid collections. In the acute stage, blood within a fluid collection may appear as simple anechoic or particulate free fluid 1. Cases have been reported where patients with simple appearing ascitic fluid on ultrasound were later diagnosed with hemoperitoneum 7, 8. Popova et al. described a patient with a traumatic fall who initially had simple ascites on ultrasound 9. Similar to our patient, a predisposing risk of bleeding from a potentially traumatic fall was known and became diagnostically valuable in conjunction with ultrasound findings. As coagulation progresses, the collection of blood becomes more hyperechoic and heterogeneous due to clotting and fibrinolysis 1. These features were described in a patient who developed iatrogenic hemothorax after the traumatic placement of a drainage catheter in a patient with a pleural effusion 10.

Our patient had a heterogeneous collection with punctiform internal echoes floating around with movement of fluid in the initial POCUS exam (Video S1), which was obtained with the probe positioned coronally in the right midaxillary line at the level of umbilicus, suggesting a dynamic exudative process (i.e. bleeding). Within an hour, we observed new fibrin strands and a gelatinous echogenic formation (Video S2) in the exact same location. In the setting of active anticoagulation, these findings made us suspect hemoperitoneum. However, the etiology was not confirmed prior to diagnostic paracentesis. A similar case in the literature reported hemothorax after a pigtail pleural catheter placement. The flow stopped abruptly during drainage, but the echogenicity of the pleural fluid increased within forty-five minutes of catheter initial insertion. The diagnosis was confirmed with insertion of a large bore chest tube, which drained 3L of frank blood 10. Similarly, our ability to diagnose hemoperitoneum was from the progression of features over time, rather than a single sonographic appearance. 

Whether unappreciated bloody ascites was present at the time of the initial abdominal CT scan cannot be known with certainty, given that the patient only started on anticoagulation afterwards. Nonetheless, the ability to conduct serial examinations with POCUS was a crucial adjunct to CT in this case. Through continuous assessments, POCUS expedited the diagnosis of worsening hemoperitoneum. This highlights a distinct advantage of POCUS over conventional imaging modalities performed in the radiology suite, where ongoing continuous monitoring is impractical.

## Conclusion

It is difficult to reliably diagnose hemoperitoneum with POCUS. The sonographic appearance of acute bleeding within a fluid space is nonspecific. In the acute stage, blood may appear as simple anechoic or particulate free fluid. As coagulation progresses, a collection of blood may become more hyperechoic and heterogeneous due to clotting and fibrinolysis. In this case, any isolated sonographic features alone would not have been diagnostic of hemoperitoneum. Instead, it was the progression of ultrasonographic features over time, in the clinical context of receiving anticoagulation, that made hemoperitoneum the most likely diagnosis. 

## Statement of Consent

Informed consent was obtained from the patient by the authors. The patient consents to the use of deidentified images, video clips, and health information published within the journal.

## Disclosures

The authors have no disclosures related to this work.

## Supplementary Materials

 Video S1

Video S2

Video S3
